# Laparoscopic low anterior resection using new articulating instruments

**DOI:** 10.1007/s10151-021-02486-9

**Published:** 2021-06-17

**Authors:** Chul Seung Lee, Yoon Suk Lee

**Affiliations:** Division of Colorectal Surgery, Department of Surgery, Seoul St. Mary’s Hospital, College of Medicine, The Catholic University of Korea, 222, Banpo-daero, Seocho-gu, Seoul, 06591 Republic of Korea

Although total mesorectal excision (TME) has become the standard surgical treatment for mid and low rectal cancers, it has certain technical hurdles. TME in men can be technically challenging, even for experienced surgeons, as extensive incisions using subtle techniques and laparoscopic instruments are required to achieve a complex anastomosis. Obtaining an effective angle, traction, and countertraction is difficult with conventional straight-fixed laparoscopic instruments. To overcome these limitations, a surgical robot system was introduced [1, 2]. This robotic system has the advantages of multi-joint mechanisms, ergonomics, and three-dimensional vision, but it is expensive. Several laparoscopic joint instruments have been introduced as alternatives to robotic systems [3]. In the attached video, we present laparoscopic low anterior resection performed by simultaneously using two laparoscopic articulating instruments.

We show the use of two articulating laparoscopic instruments (ArtiSential®, LIVSMED, Inc., Republic of Korea) which were registered as class I medical devices with the Food and Drug Administration in 2019 and have been available since November 2019 in Korea. The patient was a 62-year-old man with moderately differentiated adenocarcinoma of the mid rectum, clinical stage T3N1 and no evidence of distant metastasis. We performed laparoscopic low anterior resection with TME. The patient was placed in a modified lithotomy position, and five trocars were used: one 10-mm umbilical trocar for the laparoscope port, one 12-mm trocar on the left lower quadrant, one 8-mm trocar in the upper quadrant, and two additional 5-mm trocars used as described in Fig. 1. During the procedures, the surgeon used the two ArtiSential® instruments through the right lower and upper quadrant ports (Fig. 2). Using the 5-trocar approach, the inferior mesenteric vessels were ligated after identifying the left ureter, followed by retromesenteric dissection using a medial-to-lateral route. The splenic flexure was then mobilized, followed by laparoscopic TME dissection with preservation of the hypogastric plexus and nerves. For tumors located in the distal rectum (6 cm from the anal verge), a complete TME was performed laparoscopically after splenic flexure mobilization. The rectum was transected using an endoscopic stapler. The umbilical port was extended, and the specimen was extracted and resected with an adequate oncological proximal margin. Intracorporeal end-to-end anastomosis was performed after checking the perfusion status using indocyanine green.

**Fig. 1:**
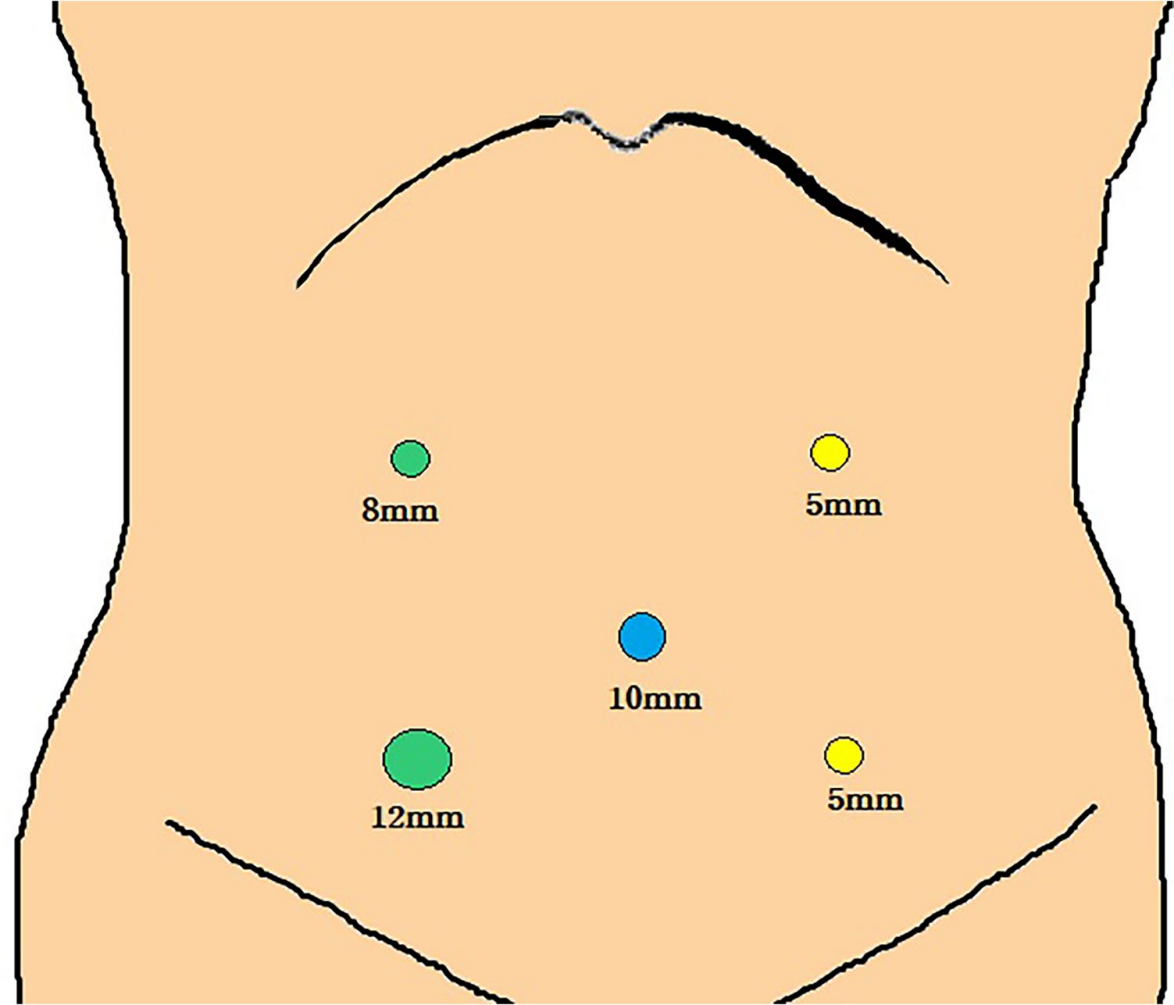
Placement of trocars

**Fig. 2:**
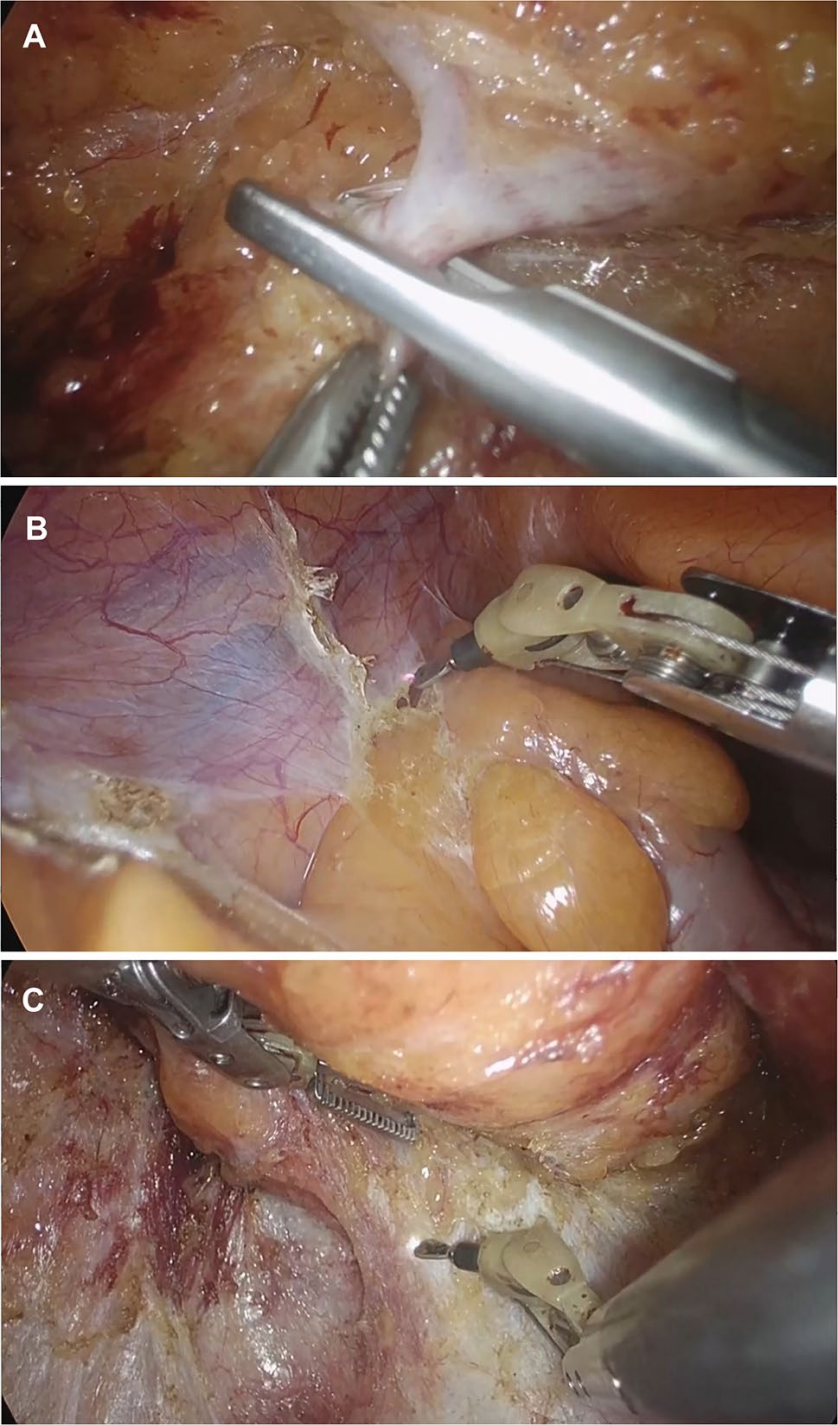
Mesenteric lymphadenectomy and total mesorectal excision. **A** High ligation of the inferior mesenteric artery; **B** lateral dissection; **C** total mesorectal excision

There were no intra- or postoperative complications. The final pathological diagnosis was pT2N0M0. Thirteen regional lymph nodes were harvested, and no metastatic regional lymph nodes were identified.

Conventional straight-fixed laparoscopic instruments have the disadvantages of reduced dexterity, limited freedom of movement, and uncomfortable ergonomics [4]. It is sometimes challenging for surgeons to obtain an effective angle, traction, or countertraction during laparoscopic surgery. Although the surgical robot system solves these problems, there is an issue of cost-effectiveness [1, 2]. A new laparoscopic joint instrument (ArtiSential®, LIVSMED, Inc., South Korea) helps surgeons easily obtain effective traction and countertraction through intuitive movements. The instrument is synchronized with the user's hand and can be moved at a 360° angle, allowing for more versatile surgical procedures than a straight-fixed laparoscopic instrument [3].

We present a standardized procedure for laparoscopic low anterior resection with TME using two new laparoscopic articulating instruments. One of the most important technical advantages of TME is the effective traction and countertraction it provides in the narrow pelvis. Unfortunately, in the presence of bulky tumors, a narrow male pelvis, or obesity, a surgical approach with conventional laparoscopic instruments is technically more challenging [5]. The use of two articulating laparoscopic instruments could be particularly helpful for exposing surgical planes and skeletonizing the primary feeding vessels.

This is the first video presenting the simultaneous clinical application of two newly released articulating laparoscopic instruments (ArtiSential®, LIVSMED, Inc., Republic of Korea). We show that laparoscopic low anterior resection using two articulating laparoscopic instruments is safe and technically feasible. These instruments are less expensive than a robot system, but like robot surgery, ergonomic and intuitive surgery is possible with multiple joints. Future comparative studies with robotic surgery are needed to demonstrate its benefits for clinical applications.

## Supplementary Information

Below is the link to the electronic supplementary material.

Supplementary file1 (MP4 200586 KB)
